# Novel Lipid-Free Nanoformulation for Improving Oral Bioavailability of Coenzyme Q10

**DOI:** 10.1155/2014/793879

**Published:** 2014-06-05

**Authors:** Huafeng Zhou, Guoqing Liu, Jing Zhang, Ning Sun, Mingxing Duan, Zemin Yan, Qiang Xia

**Affiliations:** ^1^State Key Laboratory of Bioelectronics, School of Biological Science and Medical Engineering, Southeast University, Nanjing 210096, China; ^2^State Key Laboratory of Biomembrane and Membrane Biotechnology, School of Life Sciences, Tsinghua University, Beijing 100084, China; ^3^Jiangsu Longliqi Bioscience Co., Ltd., Suzhou 215555, China

## Abstract

To improve the bioavailability of orally administered lipophilic coenzyme Q10 (CoQ10), we formulated a novel lipid-free nano-CoQ10 system stabilized by various surfactants. Nano-CoQ10s, composed of 2.5% (w/w) CoQ10, 1.67% (w/w) surfactant, and 41.67% (w/w) glycerol, were prepared by hot high-pressure homogenization. The resulting formulations were characterized by particle size, zeta potential, differential scanning calorimetry, and cryogenic transmission electron microscopy. We found that the mean particle size of all nano-CoQ10s ranged from 66.3 ± 1.5 nm to 92.7 ± 1.5 nm and the zeta potential ranged from −12.8 ± 1.4 mV to −41.6 ± 1.4 mV. The CoQ10 in nano-CoQ10s likely existed in a supercooled state, and nano-CoQ10s stored in a brown sealed bottle were stable for 180 days at 25°C. The bioavailability of CoQ10 was evaluated following oral administration of CoQ10 formulations in Sprague-Dawley rats. Compared to the values observed following administration of CoQ10-Suspension, nano-CoQ10 modified with various surfactants significantly increased the maximum plasma concentration and the area under the plasma concentration-time curve. Thus, the lipid-free system of a nano-CoQ10 stabilized with a surfactant may be an effective vehicle for improving oral bioavailability of CoQ10.

## 1. Introduction

Coenzyme Q10 (CoQ10), an essential component of the mitochondrial respiratory chain, is found in the inner mitochondrial membrane of all living cells. It is an efficient antioxidant against free radicals and lipid peroxidation [1–3]. Many studies have reported CoQ10 deficiencies among patients with cardiovascular disease [4], neurodegenerative disorders [5], diabetes [6], statin-associated myopathy [7], and cancer [8]. Supplementation with CoQ10 has proven beneficial in treating these diseases, and numerous clinical trials are investigating its use as a drug or dietary supplement [8]. However, CoQ10 is lipophilic and has extremely poor water solubility; it is known for its low bioavailability and delivery properties [9]. Thus, the empirically derived regimen for oral administration of CoQ10 takes advantage of its native lipophilic solubility and recommends coadministration with lipid-rich foods.

To improve the bioavailability of CoQ10, previously reported formulation strategies include an oil solution and suspension system [10, 11], a lipid and surfactant based emulsion [12], and a solid dispersion system [13]. However, the bioavailability of CoQ10 remained low due to the continued poor water solubility of CoQ10 in most of these formulations. Recently, much attention has been focused on lipid-based formulations including self-emulsifying or self-microemulsifying drug delivery systems (SEDDS and SMEDDDS) [9, 14] and nanoemulsion [15, 16] to greatly improve the oral bioavailability of CoQ10. Most of these formulations adopted lipid-based delivery systems, which could add significant beneficial effects to the absorption and exposure of coadministered lipophilic drugs. The unique benefits of lipids, such as their capacity to enhance water-insoluble drug solubility in the intestinal milieu, recruit intestinal lymphatic drug transport, and alter enterocyte-based drug transport and disposition, have made them very attractive candidates as carriers for oral formulations [17, 18]. In recent years, lipid-based delivery systems such as oil solutions, emulsions, or SEDDS have become popular strategies for improving the oral bioavailability of water-insoluble drugs [19, 20]. However, numerous complex factors influence the absorption from lipid-based delivery systems, including the rate of dispersion, degree of emulsification, particle size, and precipitation of drug from the formulation upon dispersion [21–23]. In addition, many studies have revealed that the lipid component of the delivery system has great influence on its ability to enhance absorption [19, 24]. Surfactants are important and necessary factors influencing drug absorption, and most lipid-based formulations have large amounts of surfactants for enhanced drug absorption [14, 19, 21]. However, high levels of surfactants may induce toxic effects, thus creating potential clinical liabilities. Additionally, the loading capacity of a drug in a lipid-based formulation is limited by its lipid solubility.

Thus, the objective of the present study was to increase the solubility and improve the bioavailability of CoQ10 by developing and characterizing a novel nanoformulation with a higher CoQ10 relative to surfactant content, while minimizing the content of surfactant to avoid potential toxic clinical effects. We developed novel CoQ10 nanoformulations that included various surfactants but no other lipids by using the established hot high-pressure homogenization (HPH) method [25].

## 2. Materials and Methods

### 2.1. Materials

Soybean lecithin (SL, Epikuron 170V) was purchased from Cargill Texturizing Solutions Deutschland GmbH & Co. KG (Germany), and CoQ10 was purchased from Zhejiang Medicine Co. Ltd., Xinchang Pharmaceutical Factory (China). D-*α*-Tocopherol polyethylene glycol 1000 succinate (TPGS) was purchased from Eastman Chemical Company (USA). Polyoxyl 40 hydrogenated castor oil (PHCO, Cremophor RH40) and polyvinylpyrrolidone (PVP, Kollidon 30) were obtained from BASF (Ludwigshafen, Germany). Polyglycerol 10 stearic acid ester (PSAE,* SWA-10D*) and sucrose palmitate (SP, P-1670) were manufactured by Mitsubishi-Kagaku Foods Corporation (Japan). Glycerol and other materials were of pharmaceutical grade. MilliQ water was used in all experiments.

### 2.2. Preparation of Nano-CoQ10 and CoQ10-Suspensions

A series of nano-CoQ10 formulations stabilized with different surfactants, such as TPGS, Cremophor RH40, SWA-10D, P-1670, Epikuron 170V, was prepared by hot HPH using a high-pressure homogenizer (model NS1001L, Niro Soavi, Italy) [25]. Briefly, 1.5 g CoQ10 powder was melted at 60°C, and 1 g surfactant was dissolved in 57.5 g glycerol aqueous solution (43.5%, w/w) at the same temperature. This solution and the melted CoQ10 were mixed together to form a crude oil-in-water emulsion at 60°C by using a high-shear mixer at 8000 rpm for 1 min. The resulting preemulsion was passed through HPH at a pressure of 1500 bar up to 6 times. The homogenization tube was cooled using a circulating water jacket. The final dispersion was cooled at ambient conditions to room temperature and stored in a sealed brown bottle at 5°C.

The CoQ10-suspension was prepared using a high-shear mixer. Briefly, 1 g Kollidon 30 was dissolved in 57.5 g glycerol aqueous solution (43.5%, w/w) at room temperature. Next, 1.5 g CoQ10 powder was dispensed into the resulting water solution to form a dispersed suspension at room temperature by using a high-shear mixer at 8000 rpm for 1 min.

### 2.3. Mean Particle Size and Zeta Potential Analyses

The mean particle size and zeta potential value of the nano-CoQ10 were determined by means of dynamic light scattering using a Malvern Zetasize 2000 (Malvern Instruments, UK). Polydispersity index was used as a measure of particle size homogeneity. Data were obtained by averaging 3 measurements at an angle of 90° in cells with 1 cm diameter at 25°C. Samples were diluted approximately 50-fold with distilled water.

To characterize the surface charge of particles, the zeta potential value was obtained by averaging 3 measurements at 25°C. Samples were diluted approximately 200-fold with distilled water.

### 2.4. Nanosystem Stability

Nano-CoQ10s were stored in a sealed brown bottle at 25°C for 180 days. Mean particle size, polydispersity index, and zeta potential of the nano-CoQ10s were analyzed at days 1 and 180.

### 2.5. Cryogenic Transmission Electron Microscopy

All samples were diluted approximately 100-fold with distilled water. For cryogenic transmission electron microscopy (cryo-TEM), 4 *μ*L samples were applied on a perforated carbon film grid (R1.2/1.3 Quantifoil Micro Tools GmbH, Jena, Germany) and blotted with filter paper (Whatman, 1 *μ*m) for approximately 3 s. After blotting, the grid was immediately plunged into precooled liquid ethane for flash freezing. The cryo-grid was held in a Gatan 626 Cryo-Holder (Gatan, USA) and transferred into TEM (JEOL JEM-2010 with 200 kV LaB6 filament) at −172°C. Samples were observed under minimal dose conditions at −172°C. Micrographs were recorded by a Gatan 832 charge-coupled device camera at a magnification of 10,000–50,000x and at a defocus of 3–5.46 *μ*m.

### 2.6. Differential Scanning Calorimetry

The initial, peak, and terminal temperatures of the reaction and the time necessary for the reaction under the static state were determined by differential scanning calorimetry (DSC) with a DSC Q2000 apparatus (TA Instruments, USA). Each sample (~10 mg) was sealed in an aluminum pan (40 *μ*L) and heated from 0°C to 80°C at a rate of 5°C/min. An empty aluminum pan was used as a reference.

### 2.7. In Vivo Study

#### 2.7.1. Animals and Operative Procedures

Animal studies were performed in the laboratory animal facility at the Life Science School of Tsinghua University, which obtained Animal Welfare Assurance from the Office of Laboratory Animal Welfare. The local ethics committee approved all animal studies performed. Male Sprague-Dawley rats (*n* = 36, 250 ± 10 g body weight) were used in the following experiments.

Silicone medical grade tubing (120 mm in length, 0.94 mm O.D. × 0.51 mm I.D., HelixMark) was used to create catheters. For insertion of the catheter in the sinus venosus, the length as measured from the tip of the catheter to the vein was set at 20–30 mm. Rats were anesthetized by intraperitoneal administration of chloral hydrate (100 mg/mL) at a dose of 250 mg/kg body weight. A longitudinal skin incision was made over the area where the right external jugular vein passed dorsally to the pectoralis major muscle. The catheter, filled with 25 units/mL of heparinized physiologic saline, was placed into the right jugular vein and then advanced into the sinus venosus. The catheter was anchored by suturing it to muscle. The free end of the catheter was passed under the skin of the dorsum of the neck just caudal to the ears and attached to the skin. Finally, the catheter was filled with heparinized saline (250 units/mL), and a metal plug was inserted into the free end of the catheter. Rats were placed in individual cages and allowed 24 h to recover from surgery with free access to food and water. At the end of the recovery period, rats were deprived of food overnight.

#### 2.7.2. Drug Administration and Blood Sampling

The next day, the catheter was flushed and filled with heparinized saline (25 units/mL). Thirty-six rats were randomly divided into the following 6 groups (*n* = 6 each): nano-CoQ10-TPGS, nano-CoQ10-PHCO, nano-CoQ10-PSAE, nano-CoQ10-SP, nano-CoQ10-SL, and CoQ10-Suspension. Formulations were administered orally to each rat with a single dose of CoQ10 (60 mg/kg).

Blood samples were drawn from the catheter using the following technique. The plunger on a syringe was retracted until a small amount of blood appeared in the needle bulb, and heparin solution was removed from the catheter together with the first sample of blood (30–50 *μ*L). The syringe was removed and replaced with another syringe for blood sample (0.5 mL) collection. This syringe was then removed and replaced with a syringe filled with saline. Blood was gently rinsed from the catheter by flushing with an equivalent volume of saline to replace the volume of blood removed. The saline syringe was removed and replaced with a syringe filled with heparinized saline. The catheter was filled with heparinized saline (50 *μ*L of 25 units/mL) and a metal plug was inserted into the catheter. Finally, the blood sample was expelled from the collection syringe into a heparinized microcentrifuge tube (1.5 mL).

After oral administration of drug, jugular vein blood samples (0.5 mL) were collected from rats and deposited into heparinized microcentrifuge tubes (1.5 mL) at the following time intervals: 0, 0.5, 1, 1.5, 2, 3, 4, 5, 6, 7, 8, 10, 12, 24, and 48 h. Blood samples were immediately centrifuged for 10 min at 4000 rpm. Plasma was collected into Eppendorf tubes and immediately stored at −20°C until used for further analyses.

#### 2.7.3. Extraction and Concentration Analyses

A mixture of plasma (0.1 mL) and an internal standard solution (0.1 mL of a 500 ng/mL methanol solution) was placed in an Eppendorf microtube. Methanol (0.8 mL) was added to precipitate proteins, and the microtube was vortexed for 1 min and centrifuged for 10 min at 12,000 rpm. The supernatant (0.7 mL) was transferred to a vial suitable for liquid chromatography/mass spectrometry (LC/MS).

The quantification of CoQ10, based on a calibration curve of CoQ10 (standard) and CoQ9 (internal standard), was determined by using LC/MS with electrospray ionization system from Agilent. The optimal settings for the MS operated in the positive ion electrospray mode were as follows: gas temperature, 350°C; drying gas flow, 8 L/min; nebulizing gas pressure, 50 psi; sheath gas temperature, 400°C; sheath gas flow, 11 L/min; capillary voltage, 4000 V; nozzle voltage, 500 V. The selected mass-to-charge (*m*/*z*) ratio transitions of CoQ10 and CoQ9 ions [*M*+1]^+^ used in the selected ion reaction were as follows: CoQ10 (863.7/197) and CoQ9 (795.6/197). The dwell time was set at 150 ms. The separation of CoQ10 was performed on an Agilent Zorbax SB-C18 rapid resolution HD (50 mm × 2.1 mm, 1.8 *μ*m particle size) with the mobile phase containing methanol, 2-propanol, and formic acid (90 : 10 : 0.1, v/v/v) at a flow rate of 0.5 mL/min over 15 min.

#### 2.7.4. Calibration and Statistical Analysis

The plasma concentration-time profile was corrected for endogenous levels of CoQ10 as follows. For each animal, the respective endogenous levels of CoQ10 at time 0 h were subtracted from the observed CoQ10 concentrations at each time point. CoQ10 plasma concentrations at different time points for individual rats were analyzed (noncompartmental analysis model) using PKSolver Professional software (China Pharmaceutical University, Nanjing, China). We calculated the area under the plasma concentration-time curve from 0 to 48 h (AUC_0–48 h_), maximum plasma concentration (*C*_max⁡_), time to maximum plasma concentration (*T*_max⁡_), and terminal half-life (*T*_1/2_). Student's *t*-tests were performed to evaluate the significant differences between the 2 formulations. All values were expressed as mean ± standard deviation (SD), and data were considered statistically significant at *P* < 0.05.

## 3. Results and Discussion

### 3.1. Physicochemical Characterization

#### 3.1.1. Mean Particle Size, Polydispersity Index, and Zeta Potential of Nano-CoQ10

Nano-CoQ10 formulations were prepared using a high-energy method with a high-pressure homogenizer. Particle size depends primarily on the pressure and the cycle time when a high-energy method is utilized to produce a nanoemulsion [26]. Each nano-CoQ10 formulation was stabilized by a surfactant with a different hydrophilic group, resulting in variable mean particle sizes as shown in Table 1. The relatively small mean particle sizes obtained are compelling with regard to enhanced bioavailability because mean particle size is a primary determinant of bioavailability for nanoformulations, and the oral bioavailability of encapsulated lipophilic compounds is increased when the size of the particles in colloidal delivery systems is reduced to the nanosize range [27, 28].

The polydispersity index indicates the quality or homogeneity of the dispersion, and a small polydispersity index (less than 0.2) indicates a narrow droplet size distribution [29]. As shown in Table 1, nano-CoQ10-TPGS, nano-CoQ10-PHCO, and nano-CoQ10-PSAE, stabilized by surfactants with a long-chain hydrophilic group, displayed similar polydispersity indices that were less than 0.2. In contrast, nano-CoQ10-SP and nano-CoQ10-SL, stabilized by surfactants with a short-chain hydrophilic group, had polydispersity indices greater than 0.2. The molecular geometry of the surfactant is one of the most important parameters influencing the homogeneity of droplets and is characterized by the packing parameter (*p*), which is the ratio of the tail group area to the head group area (*p* = *a*_*T*_/*a*_*H*_). The polydispersity index for droplets is positively correlated with the packing parameter [30].

All nano-CoQ10 formulations had negative surface charges (Table 1). Nano-CoQ10-SL was stabilized by Soybean lecithin (Epikuron 170V) as the principal emulsifier. Soybean lecithin was a mixture of phospholipids from soybean sources. Its major component was phosphatidylcholine, which was zwitterionic and neutral over a wide pH range, whereas the minor component contained negatively charged phospholipid, such as phosphatidylserine and phosphatidylglycerol, which results in the negative charge of the droplet. Other formulations of nano-CoQ10 were stabilized by nonionic surfactants, and their negative charge was likely the result of the various hydrophilic groups of their nonionic surfactants. Nonionic surfactant adsorption preferentially influences hydroxyl ions (OH^−^) on the surface of a droplet and thereby alters the zeta potential of the droplet [31].

#### 3.1.2. Stability of Nano-CoQ10

The stability tests of all nano-CoQ10s were performed at room temperature and evaluated by monitoring the mean particle size, zeta potential, and polydispersity index. In general, nanoemulsions with negative zeta potentials above −30 mV indicate stable formulations [32]. A small polydispersity index (less than 0.2) also indicates better formulation stability [29]. In our study, both the zeta potential and the polydispersity index for all nano-CoQ10s remained unaffected during the 180 days of storage at 25°C (Table 1). This stability may be due to the following points: firstly, the surfactant forms a layer around the droplets, reduces interfacial energy, and provides steric hindrance [14]; secondly, surface charges prevent nanodroplet flocculation; lastly, a small polydispersity index indicates to a great extent that the rate of Ostwald ripening is low.

#### 3.1.3. Morphology of Nano-CoQ10

**Figure 1:**
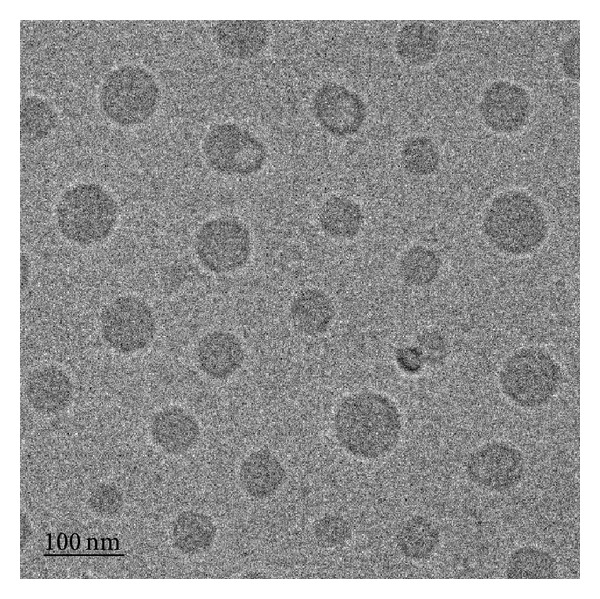
Morphology of nano-CoQ10-PHCO determined by cryo-TEM at 50,000x magnification.

The advantage of the cryo-TEM methodology is that the liquid dispersion can be frozen and viewed directly in the frozen state; thus, samples can be investigated close to their natural state [33, 34].  Figure 1 is a cryo-TEM examination of nano-CoQ10-PHCO that clearly indicates the spherical shape of nano-CoQ10.

#### 3.1.4. DSC Investigation

**Figure 2:**
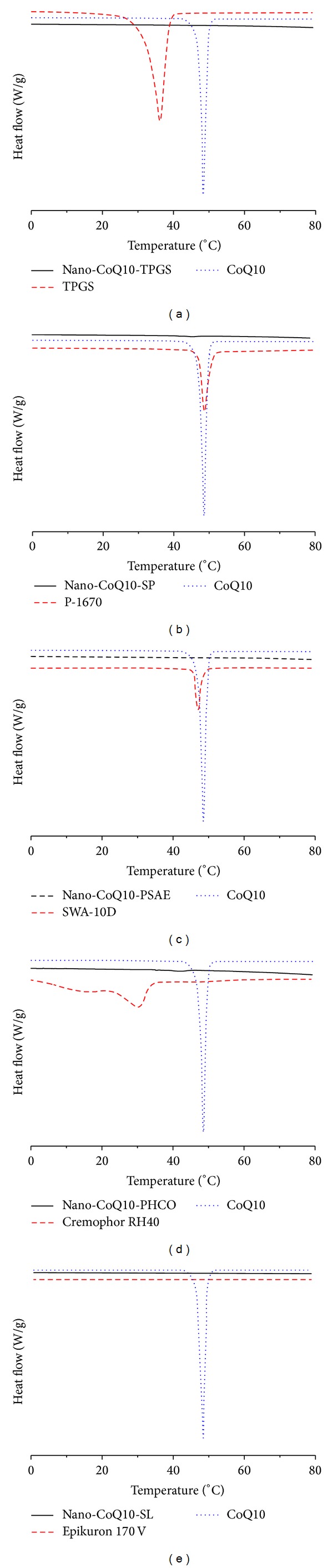
DSC curves at a heating rate of 5°C/min after 24 h storage at 8°C. (a) Bulk material CoQ10, TPGS, and nano-CoQ10-TPGS; (b) bulk material CoQ10, P-1670, and nano-CoQ10-SP; (c) bulk material CoQ10, SWA-10D, and nano-CoQ10-PSAE; (d) bulk material CoQ10, Cremophor RH40, and nano-CoQ10-PHCO; and (e) bulk material CoQ10, Epikuron 170V, and nano-CoQ10-SL.

The physical state of CoQ10 in the nano-CoQ10 formulation was investigated because it influences in vitro and in vivo release characteristics and pharmaceutical profiles. DSC curves of CoQ10, surfactant, and nano-CoQ10 are shown in Figure 2. The DSC curve of crude CoQ10 exhibited sharp endothermic peaks at 48.5°C, while that of nano-CoQ10 showed no endothermic peaks characteristic of CoQ10. The melting point of CoQ10 was absent from the heating DSC curves, indicating no heat enthalpy and a high likelihood of existing in a supercooled state. The presence of a supercooled state could be explained by the nanometer particle size having a higher specific surface area. Attributed to the Kelvin effect described by the Thomson equation [35], the nanosize effect delays or avoids the recrystallization of the CoQ10 matrix. Such supercooled nanoparticles were reported by Kuntsche et al. [36]. The supercooled state of nanoparticles may offer advantages in terms of physicochemical stability without crystallization, especially providing long-term stability at lower temperatures. However, this state is thermodynamically unstable and prone to revert to the crystalline form over time. Because preservation of the supercooled state over the shelf life of a pharmaceutical product is generally a problem, further studies will be needed to reach a definitive conclusion.

### 3.2. Oral Bioavailability In Vivo

The size of nanoparticles plays a key role in their adhesion to and interaction with biological cells. Several possible mechanisms allow particles to pass through the gastrointestinal (and other physiological) barriers. These include paracellular passage that involves particles “kneading” between intestinal epithelial cells due to their extremely small size (<50 nm), endocytotic uptake whereby particles are absorbed by intestinal enterocytes through endocytosis (particle size < 500 nm), and lymphatic uptake whereby particles are adsorbed by M cells in Peyer's patches (particle size < 5 *μ*m) [37, 38]. Lipophilic drugs generally need to be emulsified before administration. Because of their smaller particle size, nanoemulsions are reported to be particularly effective delivery systems for oral administration of bioactive compounds with several advantages over conventional emulsions, including higher optical clarity, improved physical stability, and novel rheological properties [39, 40]. In addition, pharmacokinetic studies suggest that the oral bioavailability of encapsulated lipophilic compounds is increased when the size of the particles in colloidal delivery systems is reduced to the nanosized range [27]. Therefore, to rigorously investigate the influence of surfactants on oral administration of nano-CoQ10, we fixed their particle size to less than 100 nm.

**Figure 3:**
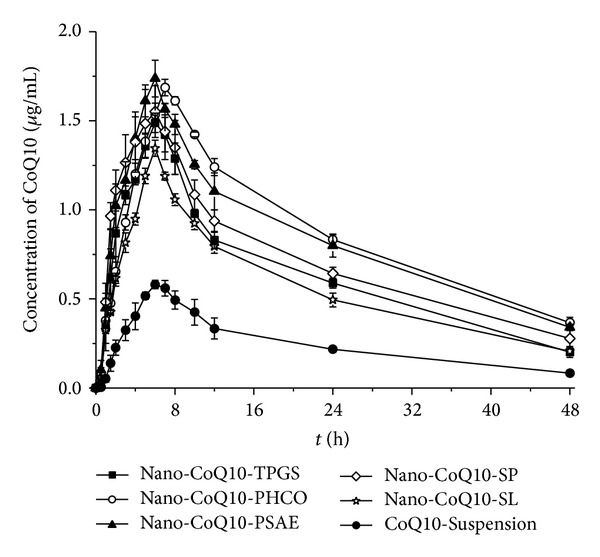
Mean plasma concentration-time profiles of CoQ10 after a single oral administration of nano-CoQ10-TPGS, nano-CoQ10-PHCO, nano-CoQ10-PSAE, nano-CoQ10-SP, nano-CoQ10-SL, or CoQ10-Suspension (60 mg/kg) in Sprague-Dawley rats (*n* = 6 per group, mean ± SD).

The pharmacokinetic profiles for orally administered nano-CoQ10s stabilized with different surfactants are shown in Figure 3 and Table 2. All nano-CoQ10 formulations significantly increased the concentration of CoQ10 in rat plasma as compared to that following administration of the CoQ10-Suspension (Figure 3). In particular, the initial plasma concentration of CoQ10 after oral administration of nano-CoQ10 was increased more rapidly and to a greater extent than that following administration of the CoQ10-Suspension. *C*_max⁡_ of CoQ10 following the administration of nano-CoQ10-TPGS, nano-CoQ10-PHCO, nano-CoQ10-PSAE, nano-CoQ10-SP, and nano-CoQ10-SL was increased by 2.5-, 2.9-, 3.0-, 2.6-, and 2.3-fold, respectively, compared to that following administration of CoQ10-Suspension; the AUC was also increased by 2.6-, 3.5-, 3.4-, 3.0, and 2.3-fold, respectively (Table 2). However, *T*_1/2_ and *T*_max⁡_ for all nano-CoQ10 modifications were not different from values obtained in the presence of CoQ10-Suspension. A nanoemulsion stabilized by salmon lecithin was reported to improve AUC_0–48 h_ (26.14 ± 3.24 *μ*g/mL × h) and *C*_max⁡_ (1.21 ± 0.12 *μ*g/mL) values of CoQ10 (60 mg/kg) after oral administration [41]. In the present study, AUC_0–48 h_ and *C*_max⁡_ values for CoQ10 after oral administration of nano-CoQ10-SL stabilized by soybean lecithin were 26.7 ± 1.13 *μ*g/mL × h and 1.35 ± 0.05 *μ*g/mL, respectively, while the greatest values of 41.02 ± 0.47 *μ*g/mL × h and 1.69 ± 0.05 *μ*g/mL, respectively, were observed after oral administration of nano-CoQ10-PHCO (Table 2). Supplementation of CoQ10 is reportedly required for several weeks to months for an observable and significant pharmacological or therapeutic effect to become apparent. The plasma threshold for the uptake of CoQ10 appears to vary by tissue type. Therefore, plasma CoQ10 concentrations need to be higher than basic plasma values to promote uptake by peripheral tissues [42].

Among the pharmacokinetic parameters assessed, *C*_max⁡_ and particularly AUC were affected by surfactants (Table 2). AUC values for CoQ10, nano-CoQ10-PHCO, and nano-CoQ10-PSAE were significantly higher than those for nano-CoQ10-SP and nano-CoQ10-TPGS. Meanwhile, the AUC value for nano-CoQ10-TPGS was significantly higher than that for nano-CoQ10-SL (*P* < 0.05). Thus, modification with surfactants enhanced the AUC of CoQ10 after oral administration in the following order: PHCO ≈ PSAE > SP ≈ TPGS > SL.

Lipophilic excipients can have significant and beneficial effects on the absorption and exposure of coadministered lipophilic drugs. After oral administration, the lipophilic drug must first dissolve within the gastrointestinal tract, a physiological and chemical barrier, before partitioning into and then crossing the enterocyte [43]. Surfactants alter the cell membrane integrity and tight junctions [43] and inhibit efflux transporters like P-glycoprotein (P-gp) [44]. The potential to attenuate the effects of intestinal efflux transporters has led to a surge in interest in the possibility of employing surfactants as permeability enhancers for drugs affecting P-gp efflux, which is a significant limiting factor in oral bioavailability [45–48]. The efflux transporter P-gp, located in the apical membranes of intestinal absorptive cells, can reduce the bioavailability of a wide range of orally administered drugs. Several surfactants have been shown to inhibit P-gp (PHCO > SP > TPGS) and thus potentially enhance drug absorption [44].

## 4. Conclusions

CoQ10 was formulated in a lipid-free nano-CoQ10 system in an attempt to increase its solubility and oral bioavailability. Nano-CoQ10 was modified with different surfactants using the hot HPH method. After oral administration in rats, lipid-free nano-CoQ10 significantly improved CoQ10 bioavailability as compared to that following administration of a CoQ10 powder suspension. We determined that surfactants were important for improving CoQ10 bioavailability. Indeed, our lipid-free nano-CoQ10s modified with different surfactants achieved similar or higher levels of CoQ10 bioavailability than that reported for a lipid-based nanoemulsion [15]. Overall, we conclude that lipid-free nano-CoQ10 formulation may be an effective vehicle for improving the bioavailability of CoQ10 and that surfactants play a key role in this improvement.

## Figures and Tables

**Table 1:** Physicochemical properties of nano-CoQ10s modified with various surfactants and the stability of nano-CoQ10s during 180 days of storage in sealed brown bottles at 25°C (*n* = 3).

	1 day			180 days		
	Particle size (nm)	Polydispersity index	Zeta potential (mV)	Particle size (nm)	Polydispersity index	Zeta potential (mV)
Nano-CoQ10-TPGS	66.3 ± 1.5	0.197 ± 0.012	−19.6 ± 1.5	72.0 ± 2.0	0.201 ± 0.01	−20.1 ± 1.0
Nano-CoQ10-PHCO	77.3 ± 2.1	0.109 ± 0.044	−12.8 ± 1.4	77.7 ± 1.2	0.117 ± 0.027	−13.5 ± 0.7
Nano-CoQ10-PSAE	89.0 ± 3.0	0.175 ± 0.014	−39.1 ± 1.1	92.0 ± 1.0	0.182 ± 0.007	−39.5 ± 0.6
Nano-CoQ10-SP	92.7 ± 1.5	0.339 ± 0.072	−37.4 ± 1.0	109.3 ± 2.1	0.297 ± 0.012	−37.5 ± 0.9
Nano-CoQ10-SL	88.0 ± 1.0	0.527 ± 0.033	−41.6 ± 1.4	102.7 ± 1.5	0.453 ± 0.063	−42.2 ± 0.8

**Table 2:** Pharmacokinetics parameters of CoQ10 in rats after a single oral administration of CoQ10 suspension and nano-CoQ10 formulations modified with various surfactants.

	*t*_1/2_ (h)	*T*_max⁡_ (h)	*C*_max⁡_ (*μ*g/mL)	AUC_0–48 h_ (*μ*g/mL × h)
Nano-CoQ10-TPGS	17.37 ± 2.56	6 ± 0	1.49 ± 0.05^⋆#^	30.38 ± 0.59^⋆#§^
Nano-CoQ10-PHCO	20.25 ± 1.23	7 ± 0	1.69 ± 0.05^⋆^	41.02 ± 0.47^⋆^
Nano-CoQ10-PSAE	21.01 ± 2.04	6 ± 0	1.74 ± 0.1^⋆^	39.81 ± 2.51^⋆^
Nano-CoQ10-SP	20.52 ± 2.78	6 ± 0	1.55 ± 0.15^⋆^	34.35 ± 2.58^⋆#^
Nano-CoQ10-SL	18.52 ± 1.42	6 ± 0	1.35 ± 0.05^⋆#§^	26.7 ± 1.13^⋆#§^
CoQ10-Suspension	18.27 ± 3.71	6.33 ± 0.58	0.58 ± 0.02	11.47 ± 0.77

(*n* = 6, mean ± SD).

^⋆^ *P* < 0.01 versus CoQ10-Suspension.

^#^ *P* < 0.05 versus nano-CoQ10-PHCO.

^§^ *P* < 0.05 versus Nano-CoQ10-SP.
